# Mindfulness-based resilience training for aggression, stress and health in law enforcement officers: study protocol for a multisite, randomized, single-blind clinical feasibility trial

**DOI:** 10.1186/s13063-020-4165-y

**Published:** 2020-02-28

**Authors:** Michael Christopher, Sarah Bowen, Katie Witkiewitz

**Affiliations:** 1 School of Graduate Psychology, Pacific University, 190 SE 8th Ave, Suite 260, Hillsboro, OR 97123 USA; 2 Department of Psychology, University of New Mexico, 2001 Redondo S Dr, Albuquerque, NM 87106 USA

**Keywords:** Mindfulness, Resilience, Stress, Law enforcement, Aggression

## Abstract

**Background:**

Law enforcement officers (LEOs) are exposed to significant stressors, elevating their risk for aggression and excessive use of force, as well as mental health consequences, including post-traumatic stress disorder, burnout, alcohol misuse, depression, and suicide. Mindfulness training is a promising approach with high-stress populations that has been shown effective for increasing resilience and improving negative mental health outcomes common among LEOs.

**Method:**

Implemented at two sites, the proposed study is designed to establish optimal protocols and procedures for a future full-scale, multisite trial assessing effects of mindfulness-based resilience training versus an attention control (stress management education) and a no-intervention control on physiological, behavioral, and psychological outcomes. To prepare for this future clinical trial, the current study is designed to: enhance efficiency of recruitment, engagement, and retention; optimize laboratory, assessment, and data management procedures; optimize intervention training and ensure fidelity to intervention protocols; and assess participant experience and optimize outcome measures across two sites. Herein, we describe the protocol and methodology of this multisite, randomized, single-blind clinical feasibility trial.

**Discussion:**

The long-term objective of this line of research is to develop an intervention that will reduce violence and increase resilience and mental health among LEOs, as well as yield significant benefits for the communities and residents they serve.

**Trial registration:**

ClinicalTrials.gov, NCT03784846. Registered on 24 December 2018.

## Background

Policing is one of the most highly stressful occupations [1, 2], and stress-impaired law enforcement officers (LEOs) are more likely to be aggressive toward suspects and use excessive force [3, 4]. Occupational stress has also been linked to disproportionately high rates among LEOs of depression and suicide [5], PTSD [6], burnout [7], and alcohol misuse [8].

Neuroendocrine markers play a significant role in physiological reactivity to stress [9, 10]. Stress responsiveness is primarily regulated by two neuroendocrine axes: the sympathetic adrenomedullary (SAM) and hypothalamic–pituitary–adrenocortical (HPA) systems. Acute psychological stress quickly activates the SAM axis, eliciting release of catecholamines such as noradrenaline and adrenaline, resulting in elevation of heart rate (HR), blood pressure (BP), and salivary alpha amylase (sAA). Dysfunction within the SAM and HPA systems among distressed and/or chronically stressed populations is thoroughly documented, including in LEOs [11, 12], and is indicated by exaggerated or blunted reactivity to stressors and/or prolonged recovery time [13, 14].

Despite elevated rates of PTSD, burnout, alcohol consumption, alcohol-related death, and suicide among LEOs, and the significant implications of compromised officers to the safety of the public, effective LEO trainings and interventions are still lacking [15]. Preliminary evidence suggests mindfulness training (MT) is a promising approach for the specific risks, challenges, and outcome patterns in the LEO population. MT has strong empirical support in lab, clinical, and community-based research, evincing outcomes such as reduced violence and aggression [16–18], and improved biomarkers of SAM and HPA stress reactivity [19–23]. MT may regulate how the individual appraises stress and increase secondary appraisals of approach-oriented coping resources, thus reducing stress reactivity [24]. Recent meta-analyses indicate that MT improves common LEO health and risk factors, including stress [25, 26], depression and suicidal ideation [27, 28], alcohol misuse [29, 30], trauma [26, 31], and burnout [32, 33], and increases psychological resilience [34, 35]. MT has been shown to be feasible and to improve health outcomes among several high-stress populations, such as military personnel [36, 37], physicians [38, 39], and firefighters [40]. Similarly, resilience training has been shown to increase the capacity to adapt to stress and improve outcomes in high-stress populations [38, 41]. Resilience training often focuses on improving skills to buffer against acute and chronic stressors, and several pilot studies have shown that LEO resilience programs improve HR, BP, sAA, and behavioral performance in live or simulated critical incident simulation tasks [14, 42–44].

The primary objective of the current study is to identify, optimize, and refine best clinical and research practices to ensure success in a future multisite efficacy trial assessing the effects of mindfulness-based resilience training (MBRT) versus an attention control, stress management education (SME), and a no-intervention control (NIC) on physiological, behavioral, and psychological outcomes among LEOs. The study’s specific objectives are as follows:

1. To enhance efficiency of recruitment, engagement, and retention
2. To ensure fidelity and equivalence of laboratory, assessment, and data management procedures
3. To optimize intervention training procedures and ensure fidelity to intervention protocols
4. To assess participant experience and optimize outcome measures

## Methods/design

### Study design and overview

The current study is a multisite randomized clinical trial assessing feasibility and across-site equivalence of all study procedures. Outcomes include physiological, behavioral, and psychological indices of officer well-being, resilience, and excessive use of force in LEOs. To assess the impact of MBRT on individual LEOs, the study will use an individually randomized group treatment (IRGT) design, randomizing individuals, not groups, to either MBRT, SME, or NIC. An IRGT design is typically used when examining an intervention that is intended to be delivered to individuals or implemented at an individual level versus a group/cluster-randomized (GR) design, which is used when the intervention is intended to be delivered and implemented at a group level [45]. MBRT is delivered in group format, but the meditation practice is implemented at an individual level. Therefore, an IRGT design is preferable over a GR design, consistent with randomized controlled trials (RCTs) examining the efficacy of mindfulness-based interventions in the extant literature [46, 47].

### Participants

LEOs will be recruited from two urban settings: Greater Portland Metro Area, OR, USA and Albuquerque, NM, USA.

### Participant recruitment

In collaboration with human resources staff at partnering police departments, we will recruit 104 LEOs (52 per site) for study participation via: 1) 10-15 min recruitment sessions, 2) emailed invitations sent to all eligible LEOs, 3) informational website, and flyers posted at department facilities, and 4) community-based police organization leadership. Recruitment will include information about the MBRT and SME trainings, assessed outcomes, confidentiality protocols, concordance between community and investigator goals, and research team contact information. Interested individuals will voluntarily contact the research team by telephone for initial eligibility screening. They will be informed of the study purpose and the randomization process, and will provide verbal informed consent for screening. Eligible LEOs will be scheduled for a second in-person screening, and if deemed eligible for the study, LEOs will then complete an individual baseline assessment.

### Participant screening and eligibility

Interested individuals will call the research offices and be read a form asking them to provide verbal consent to complete an initial eligibility screen, which will request contact, employment, and demographic information. Eligible participants must: be 21–65 years old (age limitations for participating police departments); demonstrate English fluency; be a sworn LEO at the rank of Sergeant or below; agree to random assignment to condition; and be willing to complete assessments at multiple time points and attend training groups. Individuals will be excluded from participation if they have participated in MBRT or a similar mindfulness course (e.g., mindfulness-based stress reduction). If they successfully meet these initial criteria, they are invited to the laboratory for a second in-person screening to assess for ineligibility due to severe depression, suicidal ideation, alcohol use, or PTSD, or inability or unwillingness to give written informed consent. Any prescribed medications are allowed during the course of the study. There are no required additional interventions, and there are no prohibited interventions in this study.

### General design issues

Our analytic aim is to obtain information to estimate group means, variability, and confidence intervals, identify primary and secondary outcomes, and conduct an a priori power calculation for sample size estimation for a future, fully powered, efficacy RCT. Our study is intended to optimize measurement by empirically assessing the sensitivity and responsiveness of conceptually well-justified candidate measures for the future trial. The study is not designed as an efficacy trial; as such, no primary or secondary hypotheses have been proposed regarding the impact of MBRT and SME on outcomes. However, the results from this study will be used to determine primary and secondary outcome categories for the fully powered future trial.

All of the self-report and physiological measures in this study have been validated and found to be reliable in past research. The behavioral measure that will be used is a standardized tracking system (BlueTeam) that integrates officer, administrator, and citizen data and is considered the current “gold standard” for assessing LEO use of force [48–51]. A critical objective of this study is to demonstrate the potential utility of this behavioral measure as a research outcome. A preliminary review of these data suggests there is sufficient variability in departments at both study sites; however, to our knowledge, the sensitivity to detect between-group differences and responsiveness to changes during an intervention have not yet been assessed.

The IRGT three-arm (MBRT, SME, and NIC) trial was chosen to assess the sensitivity of outcome measures to between-group differences following the 8-week training period and the responsiveness to changes in individual LEOs during the course of MBRT. The inclusion of three arms will allow us to assess the willingness of LEOs to accept randomization to these treatment options, and will enable optimization of all study procedures at multiple study sites, as well as detection and correction of any procedural flaws in order to prepare for a future, fully powered, multisite efficacy RCT.

### Sample size

Our sample size estimate is derived from a recent conceptual framework and systematic analysis for conducting a feasibility trial in preparation for the future, fully powered, efficacy RCT [52], as well as best practices [53–55] and suggestions for extending CONSORT guidelines to feasibility trials [56]. Sample sizes of 12–25 participants per arm are recommended to optimize estimation of group means and variability without oversampling in terms of diminishing returns in parameter estimation [57, 58], and to provide reasonably precise information in terms of confidence intervals around retention rates [59]. This range is consistent with the median treatment arm size of 18 found in a systematic review of feasibility trials [60], and is sufficient to assess our primary goals of optimizing study procedures and obtaining data for parameter estimation, measure sensitivity, and measure responsiveness. Given the optimization aim of our study, we did not conduct an a priori power analysis based on effect sizes and a variance inflation factor to estimate the needed sample size. Our study will provide estimates of means and variability (including intraclass correlations) that we will use for an a priori power calculation for sample size estimation in a future, fully powered, efficacy RCT.

Measure sensitivity to our study arms will be assessed by examining relative efficiency; more specifically, by dividing the *F*-value for each behavioral, physiological, and self-report outcome by the largest *F*-value among the outcomes [61, 62]. To generate the *F*-values for relative efficiency comparisons, we will conduct a one-way, between-subjects analysis of variance (ANOVA) for each outcome for MBRT versus SME and for MBRT versus NIC. To adjust for non-ignorable clustering in order to obtain more accurate (i.e., unbiased) *F*-values, we will include study site and gender as covariates in all ANOVAs (i.e., perform a one-way, between-subjects analysis of covariance for each outcome). Assessing relative efficiency entails generating *F*-values for comparison purposes and does not require determining whether the group differences are significant; however, adjusting for non-ignorable clustering will allow us to avoid deviation from a type I error rate of 0.05 and calculate *F*-values that most precisely reflect group differences for each outcome. Responsiveness to change will be assessed, in part, by comparing standardized mean responses, which will not require conducting an inferential test and therefore will not be impacted by type I or type II error rates. To assess responsiveness to change, we will also calculate partial correlations with a global impression of change measure and residualized change scores for each self-reported outcome [63], adjusting for variability due to gender and study site. Just as for our relative efficiency assessment, examination of partial correlations will not entail significance testing, but instead generating correlation coefficients for comparative purposes; however, we will partial out variability due to gender and study site to obtain precise (i.e., unbiased) estimates of covariance between a global impression of change measure and self-report outcomes.

We predict an overall attrition rate of approximately 20% across our three study arms over time. When a participant drops out of the study, we will attempt to obtain information from that LEO regarding the reason for withdrawal. Similarly, the study coordinator will contact any participants who discontinue the MBRT or SME training specifically to identify the reason for early termination (e.g., illness, not finding the training beneficial, or schedule change). In the event that a participant discontinues due to a training-related adverse event, study staff will continue to follow-up with the participant until the issue is resolved.

This information will allow us to: determine the nature of the missing data (i.e., missing completely at random, missing at random, or missing not at random) for analytic purposes; assess whether the withdrawal is due to a protocol violation, and whether the LEO experienced an adverse event due to the training or assessment; and obtain information to inform future program modifications (e.g., increase the flexibility of program delivery or reduce participant burden).

### Randomization

Using a 2:1.5:1 randomization allocation ratio for the MBRT, SME, and NIC conditions, respectively, across two sites, 47 participants will be randomized to MBRT (~ 24 at each site), 35 to SME (~ 17 at each site), and 22 to NIC (11 at each site). We will employ a permuted-block randomization procedure, stratifying by gender, to assign participants to study arms. This randomization procedure will allow us to: optimize the study procedures for use in a future, fully powered, efficacy RCT; obtain parameter estimates that reflect the causal impact of MBRT on outcomes; assess measure sensitivity and responsiveness in a way that reflects the causal impact of MBRT on participants; and ensure balance within strata and across study arms. Following baseline assessment, study staff not involved in data collection will randomly assign participants to the condition. The study statistician will create an allocation table using SPSS, and upload it into REDCap, a secure (HIPAA-complaint) Internet-based survey and data management software system housed on a secure server, which will be used to implement the randomization procedure at each site. All trial randomization codes will be stored within REDCap.

This study does not meet the criteria for a double-blinded or triple-blinded RCT, as study participants will be aware of their training assignment. To limit possible bias, participants will be asked to not reveal their treatment assignment to the data collection staff. Masking will be maintained by compartmentalizing study staff to a certain category of tasks, which will prevent the study staff involved in data analysis from interacting with study participants and knowing participant names or any other personally identifying information. Study staff involved in implementing the training will not be involved in data collection. Only study staff involved in recruitment activities will have access to both participant names and unique ID codes. The study statistician will monitor protocol fidelity around masking by maintaining contact with study staff involved in the MBRT and SME implementation, data collection, and randomization to ensure that masking is maintained and to identify any protocol violations.

### Experimental manipulation

Stress will be experimentally induced using the socially evaluative cold pressor task (SECPT), in which participants immerse their hand in ice-cold water under social threat [64, 65]. The SECPT reliably activates the SAM and HPA axes, producing elevated sAA [66] and cortisol [67, 68] levels immediately and 20 min after the stressor, respectively.

### Outcome measures

**Fig. 1:**
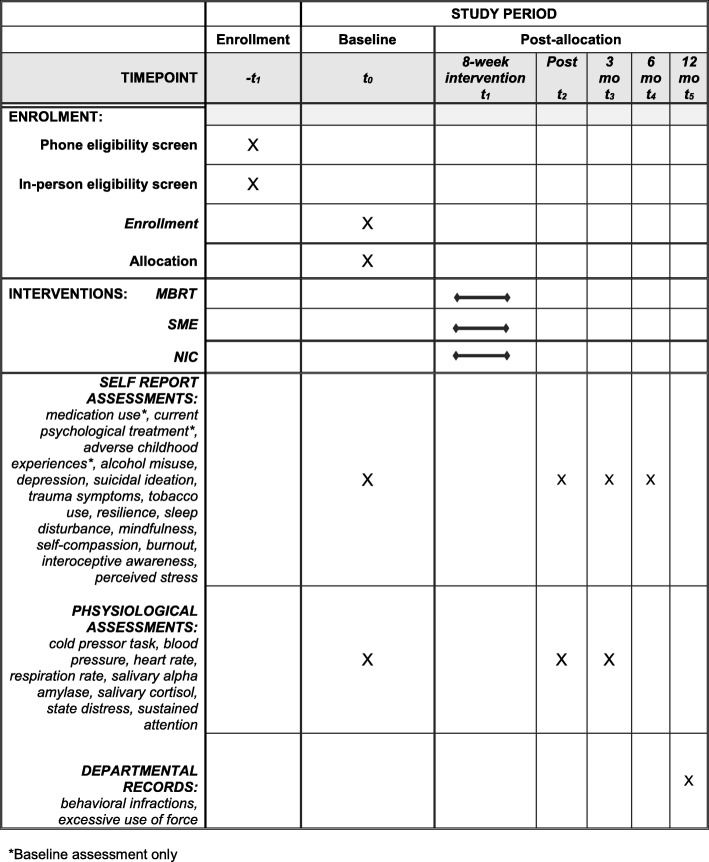
Schedule of enrollment, interventions, and assessments. *Baseline assessment only. MBRT mindfulness-based resilience training, mo months, NIC no-intervention control, SME stress management education

Assessments will be administered at baseline, post training, and 3 and 6 months following the end of the 8-week training period (see Fig. 1). The 3-month and 6-month follow-ups will occur within ± 7 days of the target date. The target date is relative to end of the training period, (i.e., 90 days after the training period for 3-month follow-up, and 180 days after the training period for 6-month follow-up).

Self-report measures include the Visual Analogue Scale [69] to assess state distress and Primary Appraisal Secondary Appraisal [70] to assess stress appraisal before, during, and after the SECPT; the Childhood Trauma Questionnaire [71]; the Expectancy/Credibility Questionnaire [72]; the Brief Resilience Scale [73]; PROMIS Alcohol Use [74] and PROMIS Alcohol Use Negative Consequences [74]; PROMIS Depression [75]; PROMIS Sleep Disturbance [76]; Concise Health Risk Tracking—Self-Report [77]; the PTSD Checklist for DSM-5 [78]; the Oldenburg Burnout Inventory [79]; the Buss–Perry Aggression Questionnaire—Short Form [80]; the Five Facet Mindfulness Questionnaire—Short Form [81]; the Self-Compassion Scale—Short Form [82]; Multidimensional Assessment of Interoceptive Awareness—II [83]; the Perceived Stress Scale-10 [84]; and Global Impression of Change [85]. Post-training satisfaction [86] and acceptability surveys assess acceptability of the training and assessment procedures for the MBRT and SME conditions. The Meditation Practice Questionnaire [87] (for MBRT) and the Stress Management Practice Questionnaire (for SME) assess MBRT and SME compliance. The iMINDr [88] mobile phone app will track compliance to assigned audio-based homework content for MBRT and SME.

The Sustained Attention to Response Task (SART) [89], a computer-based task, will measure sustained attention. Physiological measurement instruments include the Caretaker4 [90] cNIBP vital sign device to measure the heart rate (HR), respiration rate (RR), and blood pressure (BP); and the SalivaBio swab method to measure salivary alpha amylase (sAA) and salivary cortisol.

Twelve months following the end of the training period, individual-level LEO data on excessive use of force (e.g., aggressive drawing and discharge of weapons, vehicle rams, illegal takedowns, administrative and citizen complaints) will be collected from BlueTeam records [48].

### Training interventions

MBRT is an 8-week program integrating training in standardized mindfulness practices targeting factors that facilitate resilience with CBT and psychoeducation. The general curriculum structure is modeled after the mindfulness-based relapse prevention (MBRP) clinical protocol [86]. The content and language have been altered to be more relevant to LEOs, and there is emphasis on working with reactivity to stressors inherent to police work, including critical incidents, job dissatisfaction, public scrutiny, and interpersonal, affective, and behavioral challenges. MBRT will be delivered in eight weekly 2-h sessions with an extended 6-h class in weeks 1 and 7. Each session contains experiential and didactic exercises including body scan, sitting and walking meditation, mindful movement, and discussions. To supplement in-session content and support practice between sessions, the iMINDr app is programmed with audio-guided exercises and monitoring software to track daily playback. MBRT participants will also be given handouts adapted from MBRP to track daily experiences and behaviors such as triggers and stress reactions.

SME was designed as an active control condition for other mindfulness-based intervention trials [91]. It is an 8-week, 2.5-h class with weekly homework (amount matched to MBRT), one extended 6-h session, and gentle movement exercises. SME uses a group-based didactic approach with modules on physiological and dietary effects of stress, time management, sleep physiology and insomnia, nutrition, exercise, stress hardiness, and factors mitigating impacts of stress. To supplement the in-session content, and to match the amount and format of assigned homework in the MBRT condition, SME participants will also use the iMINDr app, programmed with SME-consistent audio content and playback monitoring software.

The MBRT and SME groups will be led by separate trainers, with at least master’s-level training in mental health, exercise or health science, or a related field. MBRT interventionists will have previous training in and experience with MBRT or related protocols (i.e., MBRP or other mindfulness-based interventions), and will undergo intensive training, weekly clinical supervision, and regular meetings with either or both of the multiple principal investigators (MPIs) to discuss fidelity and other clinical issues. SME interventionists will have previous experience leading health education courses, and will similarly undergo intensive training, weekly supervision, and regular meetings with expert consultants and either or both MPI.

### Outcomes and statistical analysis plan

The goal of this trial is to optimize study procedures and outcome measurement through sensitivity and responsiveness analyses. Thus, we are not grouping outcome measures into primary and secondary categories; data obtained through this trial will be used to identify primary and secondary outcomes for a future, fully powered, efficacy RCT.

### Quantitative analyses

Following best practice guidelines [92–94], including CONSORT feasibility guidelines [56] and past research [61, 95], we will measure sensitivity (using a relative efficiency approach) and responsiveness to change (examining within-group change over time). Relative efficiency analyses will be used to establish sensitivity of our outcome measures to differences between the treatment arm of focus (MBRT) and the other two arms (SME and NIC). Therefore, sensitivity analyses will focus on assessing the degree to which outcomes are sensitive to study arms, and not whether change is significant. Sensitivity will be assessed by examining the relative efficiency of each outcome variable for MBRT versus SME and NIC separately. We will calculate the relative efficiency for MBRT versus SME by conducting one-way, between-subjects analysis of covariance (ANCOVA) analyses with MBRT versus SME as the independent variable, and each outcome variable at post training as the dependent variable. We will include study site and gender as covariates. Further, we will conduct ANOVAs using the same independent and dependent variables to ensure that all inferential tests are conducted and to identify any potential type II errors among the ANCOVAs. We will then divide the *F*-statistic for each behavioral, physiological, and self-report outcome by the largest *F*-statistic among these analyses, such that the larger the number, the more sensitive that outcome is to MBRT relative to the active control group [62].

To calculate the relative efficiency of MBRT versus NIC, we will conduct one-way, between-subject ANCOVAs with MBRT versus NIC as the independent variable, each outcome variable at post training as the dependent variable, and gender and study site as covariates. We will also conduct ANOVAs using the same independent and dependent variables to ensure all inferential tests are conducted and to identify any potential type II errors among the ANCOVAs. We will then divide the *F*-statistic for each behavioral, physiological, and self-report outcome by the largest *F*-statistic among these analyses; again, the larger the number, the more sensitive the outcome is to MBRT relative to the no-treatment control group. These sensitivity analyses will allow assessment of which behavioral, physiological, and self-report outcomes are most sensitive to MBRT. When examining sAA and cortisol, AUCi will be used as the dependent variable.

To optimize measurement of stress reactivity, we will assess the HR, RR, and BP during the three phases of a stress induction task (baseline, reactivity, and recovery) as separate dependent variables. Lastly, we will examine the speed of return to baseline during the recovery phase of the stress induction task (the SECPT) for sAA, cortisol, HR, RR, and BP as additional dependent variables [96–98]. Responsiveness to change across time will be assessed in the MBRT arm for behavioral, physiological, and self-report outcomes by: calculating and comparing standardized mean responses (SMR) for outcomes by subtracting the baseline mean response for each outcome from the post-training mean response for that outcome, and dividing by the standard deviation of change for that outcome [99, 100]; and calculating and comparing partial correlation coefficients (adjusting for study site and gender) between a global impression of change measure that captures the subjective experience of changes in stress, job performance, and resilience at post training and residualized change scores (baseline to post training) for each self-report outcome [101–103]. For both SMR values and correlation coefficients, the higher the absolute value, the more responsive the outcome is to change across time in the MBRT arm. We will conduct additional correlation analyses using the same variables without adjusting for study site, gender, and interventionist to identify any potential type II errors among the partial correlations.

We will examine data for any indications of systematic site differences. Using baseline data (where sample size will be greatest) and pooling arms, since no treatment differences are expected at baseline, distributions of standardized responses on outcome measures will be summarized, graphed, and compared across sites. Key parameters, reliability estimates (i.e., Cronbach’s α), and intraclass correlations will be estimated and checked for discrepancies between sites. Identified discrepancies will be investigated to determine site-specific issues that would undermine assumptions of conceptual and structural equivalence of measurement. Given the optimization aim of our study, analyses will not adjust for intraclass correlations across study arms (and any heterogeneity in those correlations). The study will allow us to obtain information about the magnitude of intraclass correlations and the degree to which they are heterogenous across study arms, to inform the data analysis plan in a future, fully powered, efficacy RCT.

### Qualitative analyses

Focus groups will be conducted following the post-training assessment to qualitatively assess participants’ experience of all study procedures. Using an approximately 2:1:1 ratio, a random sample of 15, eight, and seven MBRT, SME, and NIC participants, respectively, will be invited to participate in two MBRT, one SME, and one NIC focus groups at each site, for a total of eight focus groups, each lasting approximately 60–90 min. Each participant will attend only one focus group. All focus groups will be conducted within 2 weeks of the end of the training period to maximize internal consistency. Per recently published guidelines for maximizing the impact of qualitative research in feasibility studies to inform an RCT [104], broad focus group question categories include: training content and delivery; trial design, conduct, and process; treatment outcomes; and measures and assessment burden.

Focus group discussions will be conducted using standardized methods, as described by Krueger and Casey [105]. Each group will be co-led by a trained moderator and an assistant, and audio recorded and transcribed. The group moderator will introduce the study and guide discussion, and an assistant will handle logistics, note preliminary themes, and assist in summarizing the discussion, sharing the summary with participants at the end of the group [105]. LEOs will be instructed to refrain from disclosing unnecessary personal information. Informed consent procedures will clearly state that participation in focus groups is voluntary, and participants will be instructed to share only what they choose to. They will be reminded that all content will be de-identified to protect their anonymity.

A thematic analysis approach [106–108] will be used to explore participant experiences with the goal of understanding the feasibility, acceptability and impact of the assessments, protocol, and training. Analyses will consist of: familiarization; initial coding; creating themes; reviewing themes; defining and naming themes; and data interpretation. Emerging themes will be used to develop a coding scheme and the team will then independently apply the codes from the finalized code structure. The coding team will meet regularly to review coding and resolve differences by in-depth discussion and negotiated consensus to ensure inter-rater reliability. A dynamic approach will be used by analyzing the first wave of focus group data following the initial MBRT and SME groups, which may result in changes to the MBRT or SME training protocol or trial procedures, and then reassessing the impact of these changes on participants’ experience of assessments, protocol, and/or training [104, 109]. We will also assess the equivalence of experience across sites, and confirm self-report measurement sensitivity based on the relationship between qualitative reports of change and quantitative results on outcome measures [110].

### Interim analyses and stopping rules

Interim analyses are not included in our protocol and will not be necessary to assess our study aims. The study will be stopped prior to its completion if: the intervention is associated with adverse effects that call into question the safety of the intervention; difficulty in study recruitment or retention will significantly impact the ability to evaluate the study endpoints (in this case, the trial will be suspended versus stopped completely to allow assessment and modification of recruitment procedures); any new information becomes available during the trial that necessitates stopping the trial; or other situations occur that might warrant stopping the trial.

### Safety assessments

Participants will be informed that MBRT and SME are considered to have very low risk for adverse events (AEs), as addressed in the consent forms. The expected minimal risks are minor aches or strains from mindful movement practices, and mild emotional distress from completing self-report measures or exposure to the stress induction procedures. To minimize risk during movement practices, participants will be instructed to recognize their physical limitations and not exceed them. Given that this is an able-bodied population, and movement practices are mild (e.g., gentle stretching), it is unlikely that injury will occur. To minimize risk related to completing the measures or procedures, participants will be notified during consent that participation is voluntary and that they can withdraw at any time. In addition to screening, suicidal ideation will also be assessed at baseline, post training, and 3-month and 6-month follow-up time points. Qualified study staff will contact any participant who endorses a suicide risk as evidenced by Concise Health Risk Tracking—Self Report (CHRT-SR) to gather more information and make an appropriate referral for mental health services.

Any data, specimens, forms, reports, audio recordings, and other records will be identified only by a participant identification (PID) number to maintain confidentiality. All study data will be stored on Box, a secure, HIPAA-compliant data storage system housed and supported by participating sites. The SalivaBio (Salimetrics, State College, PA, USA) oral swab method will be used to collect saliva. Samples will be stored at − 80 °C in a freezer-grade storage box designed by Salimetrics specifically for the cryovials until shipment. All samples from both study sites will be shipped for analysis to Salimetrics on dry ice using overnight express. All computer entry and networking programs will be done using PIDs only. Information will not be released without written permission of the participant, except as necessary for monitoring by the IRB, FDA, NCCIH, and OHRP.

Research staff will be trained to identify potential for risk and AEs. All AEs will be documented using an adverse event form, classified by severity level and relatedness to the study, and stored in REDCap. Minimally, the MPIs will be contacted in the event of any AE. The MPIs will review the list of AEs with the Independent Monitoring Committee (IMC) on a semi-annual basis. The IMC is composed of a biostatistician and two clinical psychologists with combined experience with clinical trials, mindfulness-based interventions, and adaptations of clinical protocols for underserved and high-stress populations. The IMC members are not associated with this research project, are not part of the key personnel involved in this grant, and have not collaborated with the two MPIs, within the past 3 years. The members of the IMC are qualified to review the patient safety data generated by this study. The IMC will serve in accordance with the guidelines set forth in a charter provided to each member. Serious adverse events that are unanticipated, serious, and possibly related to the study training will be reported to the IMC, IRB, and NCCIH in accordance with requirements. Protocol modifications will be approved by the NCCIH and the Pacific University IRB.

Weststat, an employee-owned NCCIH contract research company, will provide external oversight of the study. Following NCCIH protocol approval, Weststat schedules a 1-day site initiation visit prior to beginning participant enrollment. A second 2–3-day interim visit occurs after 50% of the participants have been enrolled in the study or 1 year after initiation (whichever comes first). Weststat schedules a final close-out visit after the last participant has competed the study.

## Discussion

Excessive LEO use of force in critical incident encounters is one of the most divisive human rights issues in the United States [111–113]. Substantial personal, social, and economic costs of LEO stress suggest a clear need for innovative and novel preventive intervention programs to reduce aggression and excessive use of force, mitigate stress reactivity, and improve LEO mental health. This need is further highlighted by a recent meta-analysis of existing research on stress management interventions among LEOs, which found no significant impact on negative physiological, psychological, or behavioral outcomes [15]. Although there is a growing literature on interventions for high-stress populations, such as military and medical professionals, research on LEOs, representing one of the highest-stress occupations, remains sparse.

Mindfulness-based interventions have shown efficacy in diverse populations, affecting varied psychological and health outcomes [25, 27, 114]. Preliminary evidence suggests that mindfulness training is a promising approach for the specific risks, challenges, and outcome patterns present in the LEO population, with strong empirical support in laboratory-based, clinical, and community-based research. Mindfulness training has been shown to be feasible and to lead to improved health outcomes among several similarly high-stress populations, such as military personnel [36, 115], physicians [39, 116], and firefighters [117]. Resilience training has also been shown to improve the capacity to adapt to stress and improve outcomes in high-stress populations [41, 118]. Based on work with military and first-responder populations, Jha et al. [119] recently proposed a conceptual model of risk reduction among high-stress professions, in which mindfulness and resilience synergistically impact health and risk factors, such as those common among LEOs. This research, along with our published pilot data [120, 121], lay the groundwork for the current study, which adapts established evidence-based mindfulness training to optimally target specific resilience-related physiological, psychological, and behavioral processes that are often problematic in the LEO population.

This trial represents the first multisite research study focusing on officer health, aggression, and violence in a law enforcement population. To inform recruitment and training procedures, officers are included amongst the investigator team, alongside experts in mindfulness training, psychophysiology, and data analytic methods. The study lays groundwork for a future, fully powered, multisite trial assessing the timely and understudied potential of mindfulness-based training for law enforcement officers. Specifically, the current study will evaluate appropriateness of measures and procedures, fidelity of the MBRT and SME training and assessments procedures across sites, and acceptability of all procedures by participants.

## Trial status

The trial has been registered on ClinicalTrials.gov (December 2018), and all study procedures have been approved by the Single Institutional Review Board at Pacific University. Protocol version 11.0; protocol version date, December 15, 2019. Research staff and trainings in the delivery of MBRT and SME have been completed at both study sites, and all equipment and data software systems have been set up and tested. Participant recruitment and enrollment for the first of two cohorts began in October 2019. Baseline assessments began in January 2020. We anticipate final participant assessment data to be collected by March 2021.

### Dissemination plan

The authors will ensure that results from the clinical trial are submitted to ClinicalTrials.gov as outlined in the “NIH Policy on the Dissemination of NIH-Funded Clinical Trial Information” and according to the policy’s specific stated timelines. Informed consent documents for the clinical trial will include a specific statement relating to posting of clinical trial information on ClinicalTrials.gov. Pacific University has an internal policy in place to ensure that clinical trial registration and results reporting occur in compliance with NIH policy requirements. All investigators are aware of and agree to abide by the principles for sharing research resources, as described by the NIH in “Principles and Guidelines for Recipients of NIH Research Grants and Contracts on Obtaining and Disseminating Biomedical Research Programs.”

We are also committed to disseminating the data and study findings in a wide and efficient manner. Data generated from the proposed study will be presented at national or international conferences and published in a timely fashion. All final peer-reviewed manuscripts that arise from this proposal will be submitted to the digital archive PubMed Central. Wherever applicable, data will be deposited to appropriate public repositories. Key audiences for dissemination of results include psychologists and other mental health professionals, law enforcement officers and agencies, and academic researchers.

## Supplementary information

**Additional file 1.** SPIRIT 2013 Checklist: Recommended items to address in a clinical trial protocol and related documents.
